# A Highly Versatile Polymer Network Based on Liquid Crystalline Dendrimers

**DOI:** 10.3390/ijms22115740

**Published:** 2021-05-27

**Authors:** Ramón Cervera-Procas, José-Luis Serrano, Ana Omenat

**Affiliations:** Instituto de Nanociencia y Materiales de Aragón (INMA), CSIC-Universidad de Zaragoza, Pedro Cerbuna 12, 50009 Zaragoza, Spain; rcerverap@gmail.com

**Keywords:** dendrimer, polymer network, liquid crystal, mechanical properties, microporous structure, responsive materials, dye encapsulation

## Abstract

Highly functional macromolecules with a well-defined architecture are the key to designing efficient and smart materials, and these polymeric systems can be tailored for specific applications in a diverse range of fields. Herein, the formation of a new liquid crystalline polymeric network based on the crosslinking of dendrimeric entities by the Cu^I^-catalyzed variant of the Huisgen 1,3-dipolar cycloaddition of azides and alkynes to afford 1,2,3-triazoles is reported. The polymeric material obtained in this way is easy to process and exhibits a variety of properties, which include mesomorphism, viscoelastic behavior, and thermal contraction. The porous microstructure of the polymer network determines its capability to absorb solvent molecules and to encapsulate small molecules, like organic dyes, which can be released easily afterwards. Moreover, all these properties may be easily tuned by modifying the chemical structure of the constituent dendrimers, which makes this system a very interesting one for a number of applications.

## 1. Introduction

In recent years, growing interest is being observed in the development of smart materials, which are able to respond to external stimuli and result in a change of their size or shape. Materials such as hydrogels [1,2], conducting polymers [3], dielectric [4], and liquid crystalline elastomers [5,6,7] etc. responding to pH changes, solvent composition, temperature, electric and magnetic fields, and light have been reported. Generally speaking, polymers play an important role in this field because of their flexibility, light weight, and low costs. In particular, the liquid crystalline elastomers are interesting as soft actuators as a result of their unique nature that combines the anisotropic characteristics of liquid crystal phases and the rubber elasticity of polymer networks [8,9,10,11,12,13,14,15]. This ability to change the shape reversibly upon application of external stimuli makes them good candidates for applications as sensors and actuators, as predicted by de Gennes in 1975 [16].

Dendrimers and dendrimeric units (dendrons) may be considered as polymers with geometrically-restricted structures, and for this reason, they are one of the most versatile, compositionally and structurally controlled synthetic nanoscale building blocks available today [17,18,19,20]. The combination of the structural properties of dendrimers with those of liquid crystals, such as directionality—anisotropy—and self-assembly, gives rise to new materials (the so-called dendromesogens) with singular characteristics [21,22,23,24].

There are only a few examples of dendrimeric networks described up to now. Tomalia et al. reported on the assembly of dendrimers that can be statistic or structure controlled, the so-called megamers [25]. Dvornic et al. developed several polymeric systems based on poly(amidoamine-organosilicon) (PAMAMOS) networks [26] and more recently, have described a material formed by the crosslinking of a polyamidoamine-polyethylene glycol (PAMAM-PEG) dendrimer [27]. Our group described the preparation and liquid crystalline properties of a network based on poly(propyleneimine) dendrimers with chiral non-mesogenic units [28] and the preparation of a polymer network by the thiol-yne photocrosslinking of a liquid crystalline azo-containing dendrimer [29]. J. Sun [30] reported the preparation of vinyl-terminated liquid crystalline dendrimers based on dendritic polyols and their siloxane-based elastomers. This is quite an unexplored topic of research up to now, and therefore, the properties found for these materials open a broad field of possibilities for further investigation.

The microporous structure of the polymer networks determined their capability to absorb solvent molecules and to encapsulate small molecules, such as organic dyes, which can be easily released afterwards. The microgels obtained by the swelling of the polymer networks may be applied as carriers of drugs, as diagnosis agents, microreactors, and for the synthesis of nanoparticles [31].

The synthetic strategy developed in the preparation of the dendritic networks is based on the crosslinking of reactive dendrimers. The dendrimeric networks are formed via cross-linking using the Cu^I^-catalyzed variant of the Huisgen 1,3-dipolar cycloaddition of azides and alkynes to afford 1,2,3-triazoles [11,32,33].

**Figure 1:**
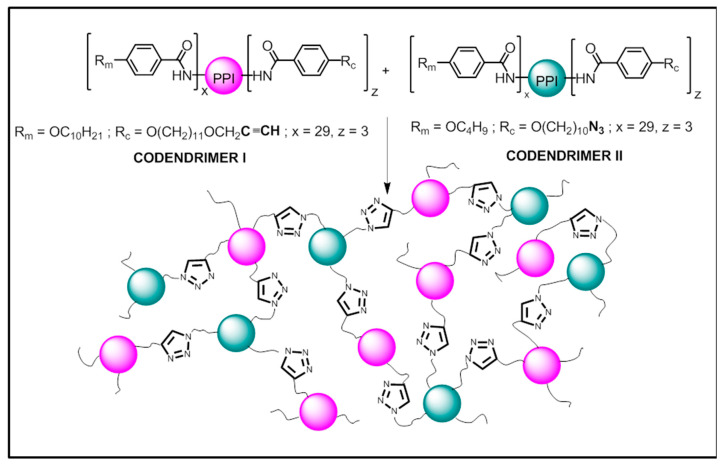
Schematic representation of the codendrimer precursors I and II and the dendrimeric network obtained thereof.

Here, we report on the preparation and characterization of materials based on a new polymer system consisting in a liquid crystalline network, which is formed by the covalent union of discrete dendritic entities (codendrimer I and codendrimer II) as shown in Figure 1.

The mesogenic, mechanical, swelling, and encapsulating properties of the polymeric network prepared in this manner have been investigated.

## 2. Results and Discussion

### 2.1. Synthesis

The commercially available fourth generation of the poly(propyleneimine) dendrimer (PPI) containing 32 –NH_2_ terminal groups was functionalized on its periphery by the introduction of promesogenic units, namely 4-n-alkyloxyphenyl moieties. Depending on the number and length of the terminal alkyloxy chains on the phenyl rings, the dendrimers obtained displayed different mesophases [34]. Moreover, a small number of peripheral units must contain the reactive terminal groups, i.e., azide or alkyne, which are used to covalently bind the discrete macromolecules to yield the dendrimeric networks (Figure 1). Full experimental details are described below.

### 2.2. Thermal Properties

Codendrimers I and II and the dendrimeric network were analyzed by thermogravimetry and the results showed that they are thermally stable up to 200 °C, at which a slow weight loss started. The maximum of the first derivative of the weight loss occurred at ca. 350 °C for all the materials.

**Figure 2:**
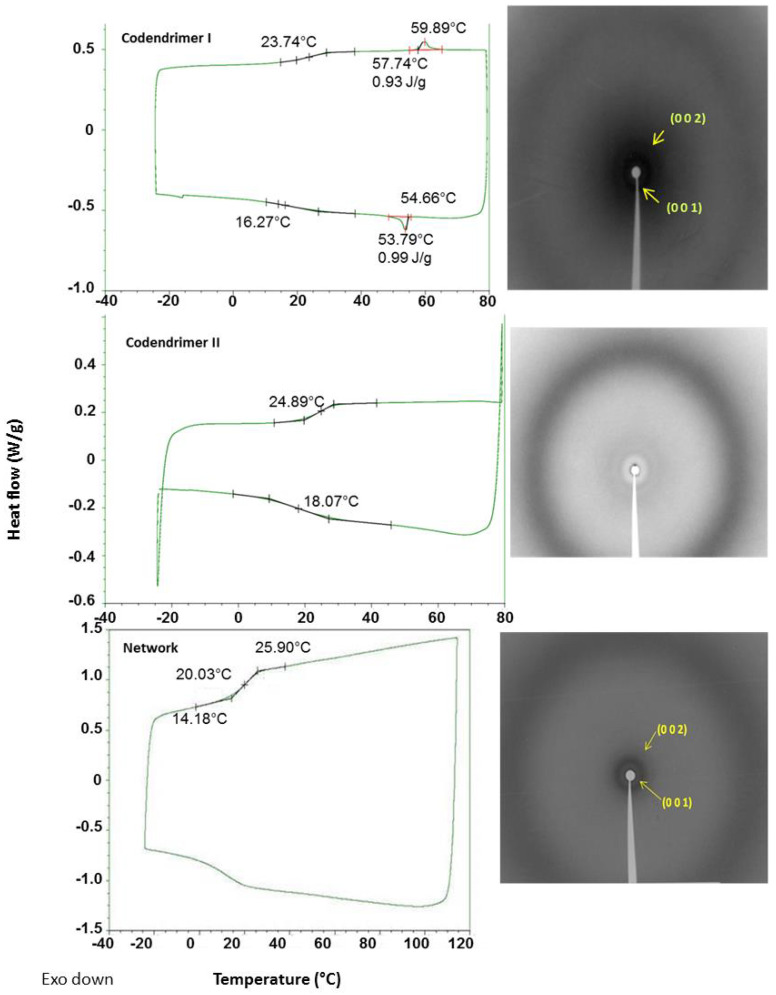
DSC thermograms (**left**) and X-ray diffractograms (**right**) of the codendrimer I, the codendrimer II, and the dendrimeric network. The XRD studies were performed at 20 °C on samples previously heated up to 40 °C and cooled down to room temperature.

As shown in Figure 2 and Table 1, all compounds are amorphous materials exhibiting glass transitions at temperatures around 25 °C. In addition, codendrimer I and the dendrimeric network showed a smectic A mesophase, which was identified by polarized optical microscopy (POM) and X-ray diffraction (XRD). Codendrimer II showed no mesomorphism and it presented a transition from the glassy state to the isotropic liquid. In both cases, the small amount of functionalized terminal units (azide and alkynyl units) were incorporated in the structure without producing significant changes in the mesomorphic behavior of the codendrimers.

Under the microscope, the dendrimeric network was observed as birefringent material. This birefringence was lost at temperatures above 80 °C and reappeared upon cooling, marking the transition to the isotropic state. Some residual birefringence was observed in the isotropic phase, indicating that a certain degree of order and a weak orientation of the network were maintained as a consequence of the crosslinked nature of the polymeric material. The mesophase of the dendrimeric network was determined to be smectic A by X-ray diffraction. The measured layer thickness for the network was 42.4 Å at room temperature. It is noteworthy that the crosslinking of a smectic A dendrimer with a non-mesogenic one yielded a network with improved mesogenic properties (broader smectic A range), thus a beneficial effect of the network on the mesomorphism of the material may be inferred.

**Figure 3:**
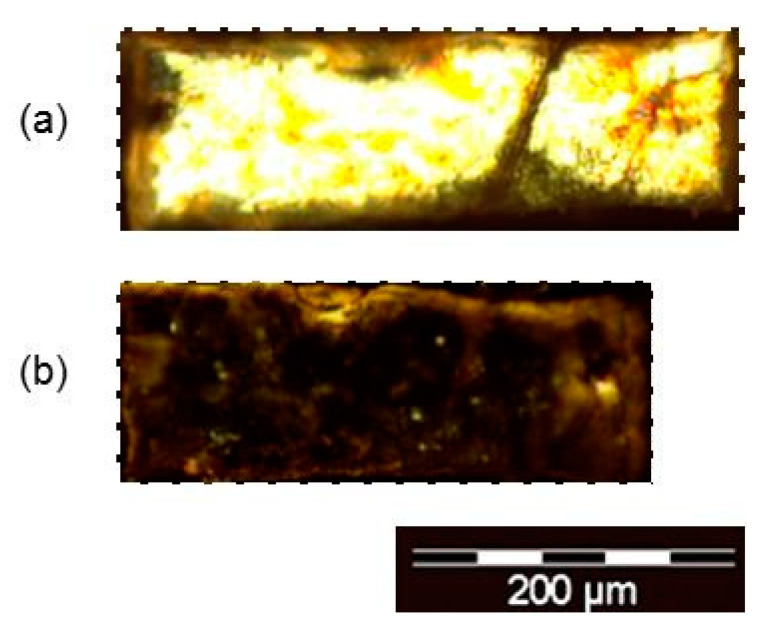
Microphotographs of the dendrimer network at (**a**) room temperature in the SmA mesophase and (**b**) 80 °C in the isotropic phase.

Films of the dendrimeric crosslinked networks underwent anisotropic thermal contraction upon heating from the liquid crystalline to the isotropic phase, as a result of the loss of the orientational order of the mesogens within the network in the liquid crystalline phase, and the consequent adoption of a spherical conformation in the isotropic phase. This phenomenon was observed in the microscopic scale and a contraction of ca. 14% is calculated by measuring the lengths of the dendrimeric network sample (Figure 3).

### 2.3. Mechanical Properties

**Figure 4:**
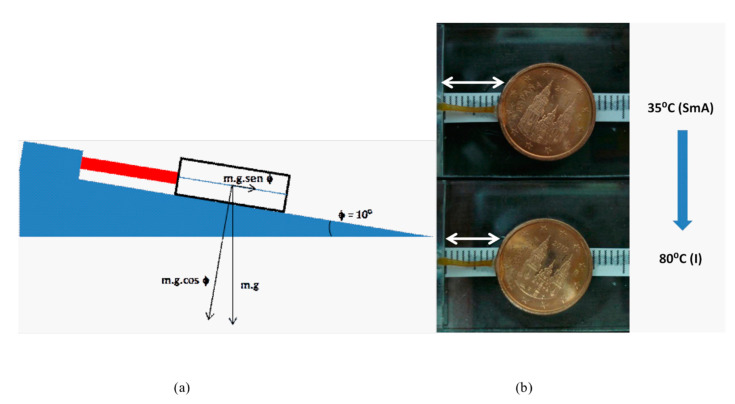
(**a**) Schematic representation of the experiment designed to measure the mechanical worked performed by the dendrimeric network under study. (**b**) Mechanical work performed by the network upon thermal contraction. Photographs of a dendrimeric fiber at 35 °C (SmA phase) (above) and contraction observed at 80 °C (isotropic phase) (below).

In order to demonstrate the macroscopic scale contraction of a fiber of the dendrimeric network and its capability to perform a mechanical work, we reproduced the experimental setup described by Percec [35], in which the thermal contraction experimented by a dendrimeric fiber lifts a coin on the inclined plane of a Mettler hot stage (Figure 4).

A dendrimeric fiber (length = 9 mm, mass = 10 mg) was stuck to a glass plate by one end and to a coin by the other (mass = 2305 mg). The system was mounted on a Mettler FP 82 stage with an inclination of 10°.

The fiber suffered a thermal contraction of ca. 10% when passing from the SmA phase to the isotropic phase. The work performed by the fiber upon contraction can be calculated as:

P = m·g = 2.305·10^−3^ Kg·9.81 m/s^2^ = 2.3·10^−2^ N;

F_mat_ = m·g·sen10°; 

F_mat_ = 2.305·10^−3^ Kg·9.81 m/s^2^ · sen10° = 3.9·10^−3^ N ~ 390 milligram-force;

W_mat_ = 3.9·10^−3^ N·10^−3^ m = 3.9·10^−6^ J.

Since the mass of the fiber is 10 mg, we observe that it is able to perform a force of, at least, 39-times its weight.

**Figure 5:**
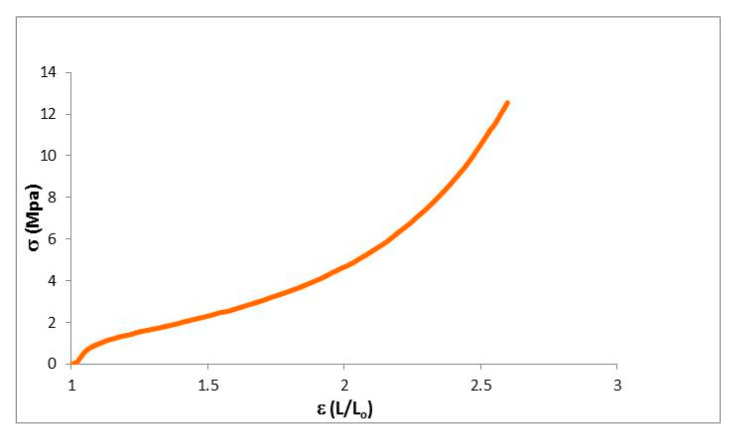
Stress–strain curves of the dendrimeric network.

The mechanical properties of the dendrimeric network were further characterized by measuring their stress–strain curves on samples prepared with dimensions in the following range: 6.60–7.60 mm length, 2.5 mm width and thickness of 0.15 mm (Figure 5). When the strain was just applied, the network responded as an elastic material up to a deformation of 3–5% with a steep increase of the stress. In Table 2, the values of Young’s modulus and the ultimate properties are summarized. After this initial steep increase, the gradient of stress reduces, mimicking the behavior of a conventional elastomer with a rubber plateau followed by an up-turn at high strains. The material is able to absorb a great part of the strain energy. The applied tension reorganizes the structure of the material by lengthening the dendrimer matrices and the alkyl chains. When the applied strain became greater and the deformation of the material was around 150%, it started to break. The maximum tensile strength for the network was 12.7 MPa.

### 2.4. Microstructure and Encapsulation Studies

**Figure 6:**
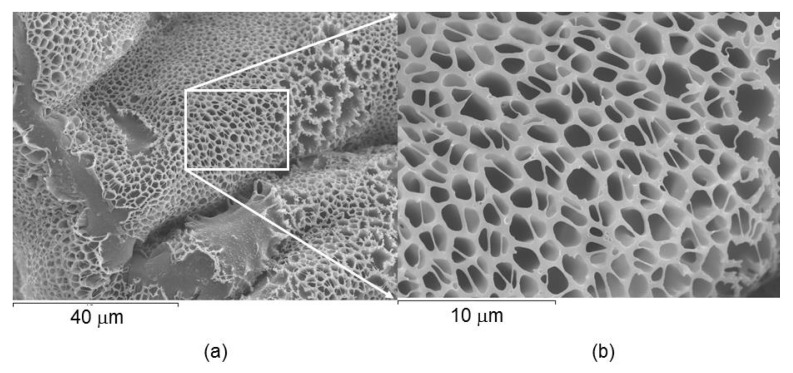
(**a**) SEM microphotographs of the dendrimeric network deposited as a dry film at room temperature. (**b**) Enhanced image corresponding to the square in (**a**).

The microstructure of the dendrimeric network was studied by SEM at room temperature. As shown in the microphotographs in Figure 6, obtained from dry films, the dendritic network presented a microporous structure, with a porous size between 0.5 and 2 μm.

#### 2.4.1. Sorption of Solvents

The porous nature of the network led us to investigate its ability to capture several solvents, behaving as a microsponge. For this study, a sample of a known weight of the dendrimeric network was immersed in 2 mL of different solvents during 60 h and the swelling ratio (SR) was calculated taking into account the following formula:

SR = Ws/Wd(1)

where W_s_ is the weight of the solvent in a swollen network and W_d_ is the weight of the dry network. All the experiments are performed at room temperature. The results obtained are gathered in Table 3.

**Figure 7:**
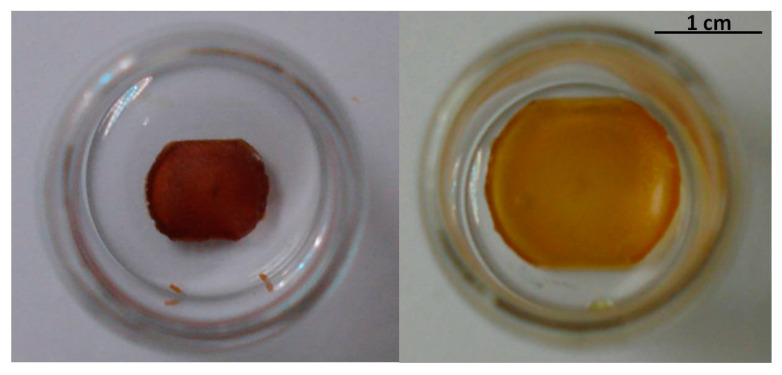
Photographs of the dry (**left**) and swollen (**right**) dendrimeric network with THF.

Non-polar aprotic solvents such as dichloromethane and tetrahydrofuran presented the highest swelling capacity (swelling ratios of 9.8 and 8.6, respectively), due to their chemical affinity with the dendrimeric network. In the case of ethanol, a moderate degree of swelling was observed (swelling ratio of 2.0), whereas with water and hexane, almost no effect occurred, and the increase of the sample weight was due to the wetting of the surface of the polymer film rather than to the penetration of the solvent within the pores of the network. These results confirm the active role of the dendrimer in the encapsulation process. In Figure 7, the significant increase of size of the network when swollen with THF is shown.

#### 2.4.2. Encapsulation of Dyes

We carried out some preliminary experiments in order to check the usefulness of our porous polymer network as a solid medium to host a laser dye. We tested three organic dyes with a diverse chemical nature: Rhodamine B (Rh-B) neutral, cationic or zwitterionic depending on the pH of the medium, Disperse Red-1 (DR1) polar, and β-carotene (βC) hydrophobic and apolar, since it has been proved that trapping organic dyes into solid matrices is a convenient alternative to overcome some problems found in the use of dyes in solution, such as the poor thermal stability or the volatility of the solvents.

The general method to carry out the experiments of encapsulation and release of the dyes in and from the network is described in the section Materials and Methods.

##### Rhodamine B

**Figure 8:**
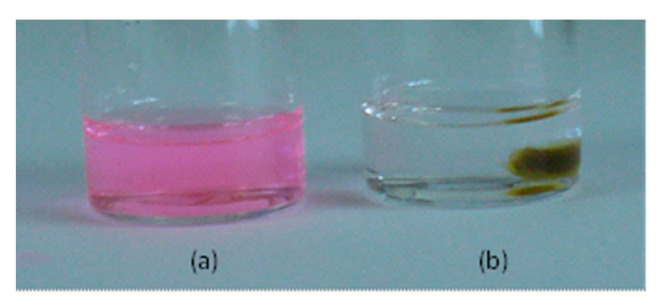
Photographs of (**a**) a 10^−5^ M solution of Rh-B in THF; (**b**) After introducing a piece of the dendrimeric network (10 mg).

It is well known that the fluorescence emission intensity of Rh-B is highly dependent of the pH and the host medium [36,37]. For example, when Rh-B is solved in a non-polar solvent such as THF, the maximum fluorescence intensity falls rapidly with increasing pH value. The presence of the amino-rich dendrimeric network in a solvent produced the corresponding increase of its pH. In fact, when a sample of the dendrimeric network was immersed in a colored solution of Rh-B in THF, this became immediately colorless due to the increase of the pH (Figure 8). However, in a polar solvent such as ethanol, the maximum fluorescence emission intensity was not significantly affected. Depending on the pH of the ethanolic solution, Rh-B presents the following maxima absorption and emission [λ_a_ = 552 nm, λ_e_ = 577 nm at acidic pH; λ_a_ = 543 nm, λ_e_ = 563 nm at basic pH]. For this reason, although the swelling ratio found for THF was higher than that of ethanol, we used solutions of Rh-B in ethanol to carry out the encapsulation experiments with the dendrimeric network under study. The pH of a solution of Rh-B in ethanol (c = 10^−3^ M) was measured before and after being in contact with the dendrimeric network and the values obtained were 4.4 and 7.3, respectively.

**Figure 9:**
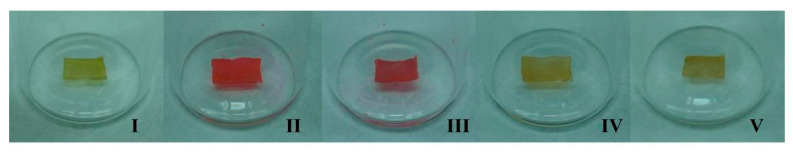
Photographs of (**I**) dry network; (**II**) swollen network with Rh-B/ethanol; (**III**) dry network with encapsulated Rh-B; (**IV**) swollen network with ethanol, after Rh-B release; (**V**) dry network.

Rhodamine B was embedded in the network following the experimental procedure explained below. In Figure 9, a series of photographs of the whole process is shown. This procedure is repeated with three solutions of Rh-B in ethanol of different concentrations. Table 4 gathers the results obtained.

From these results, it can be established that the concentration of the solution of Rh-B absorbed by the network was approximately 2.5 times greater than that of the initial solutions used in all the three cases investigated. The chemical affinity of Rh-B with the dendritic structure led to this deviation from the equilibrium state, in which the concentration of the solution in- and outside the network would be the same. However, this affinity is not as high as to give rise to an irreversible binding of the dye to the dendrimer, since we have verified that it was completely released from the network when this immersed in pure ethanol.

Once we confirmed that the dendrimeric network is able to encapsulate Rh-B within its porous structure, the absorption and emission spectra of the dye-doped polymer film were measured and the values obtained are λ_a_ = 552 nm and λ_em_ = 580 nm.

**Figure 10:**
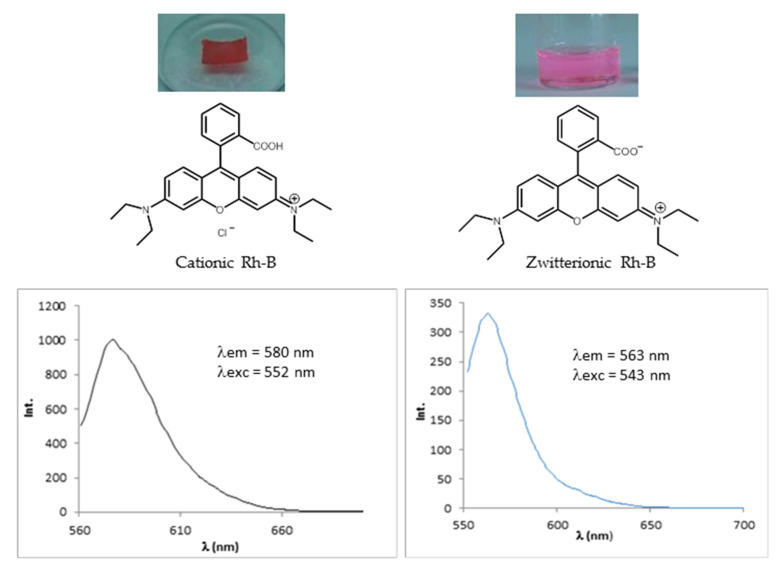
Effect of the interaction between Rhodamine B and the PPI-derived dendrimeric network in the molecular form of the dye (cationic when embedded in the network and zwitterionic when released from the network).

To determine the amount of Rh-B that was embedded in the network, the film was immersed in ethanol to release the dye. The network was dried and the absence of Rh-B was confirmed by measuring its UV-vis absorption and fluorescent emission. The amount of dye was quantified by UV-vis spectroscopy in solution and the result was 27.9 μg of Rh-B. Taking into account that the dimensions of the polymer film were 6 × 4 mm^2^ and neglecting its thickness since the fluorescent emission is measured in a reflection mode, the concentration of Rh-B in the polymer network was ~1.16 μg/mm^2^. The maximum absorption and emission wavelengths of the released Rh-B in ethanol was λ_a_ = 543 nm and λ_e_ = 563 nm, respectively, as correspond to a basic ethanolic solution of Rh-B. Thus, we can conclude that Rh-B presented its cationic form when was embedded in the dendritic PPI-based network, whose amino groups deprotonated the carboxylic acid of the cationic form of the Rh-B giving rise to its zwitterionic form when released from the network and solved in ethanol (Figure 10).

##### Disperse Red 1 and β-Carotene

Disperse Red 1 is a polar azoic dye, which is used in nonlinear optics and is incorporated into aromatic polymers to obtain model electrooptic polymer films.

β-Carotene is an apolar and hydrophobic dye. For both dyes, their absorption and emission spectra in the visible are not pH-dependent in tetrahydrofuran and thus, the experiments of encapsulation/release were carried out in this solvent, which swells the network much more than ethanol.

The results obtained for the three dyes under study are gathered in Table 5, and demonstrate that the dendrimeric network possesses a microsponge morphology, in which different types of organic molecules may be embedded, and that this is a reversible process. Depending of the intermolecular interactions, the dendrimeric network plays an active role in the encapsulation process. In this way, the interactions between the network and Rhodamine B are strong and, thus, the concentration of the dye in the network was higher than that of the initial solutions employed. In the case of Disperse red 1, the interactions are weaker, and the concentration of the dye in the network was just slightly higher than that of the initial solution. For β-carotene, no significant interactions between the network and the dye exist, and the concentration of dye adsorbed was practically that of the initial solution.

## 3. Materials and Methods

### 3.1. Synthesis and Characterization of the Codendrimers

**Figure 11:**
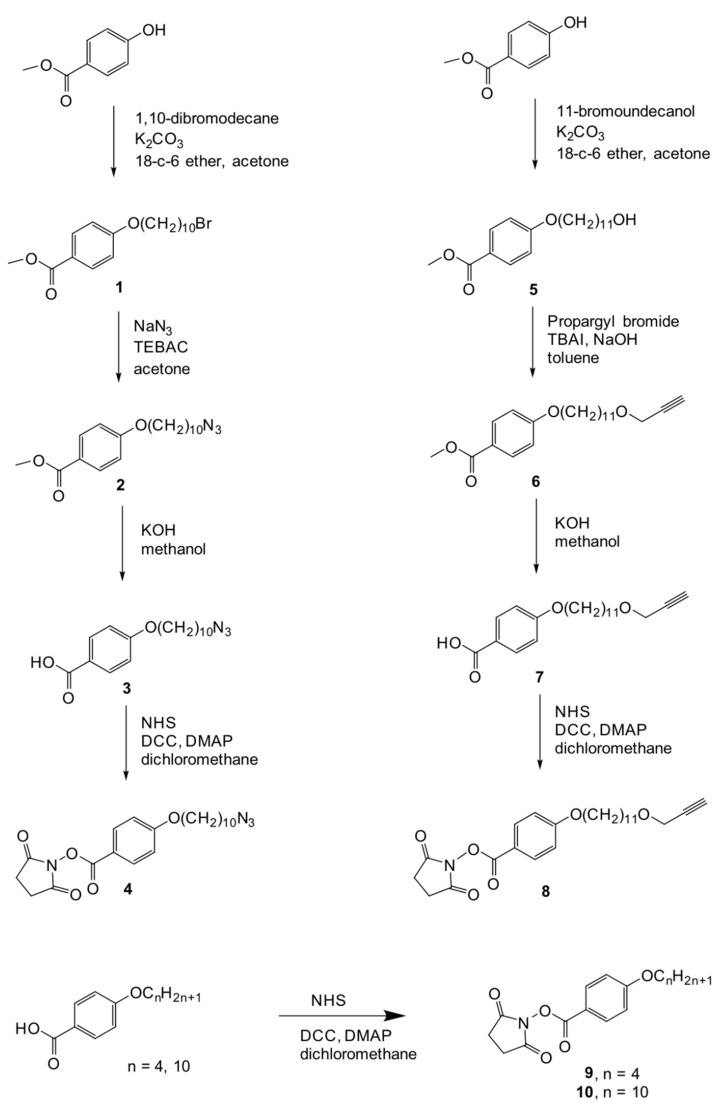
Synthetic route to the functional units **4**, **8**, **9**, and **10**.

The synthesis of the codendrimers is shown in Figure 11. The codendrimers were prepared by reaction of the fourth generation PPI dendrimer with the corresponding activated promesogenic units in the stoichiometry established to render the final codendrimers, which have the peripheral units attached via amide linkages. The codendrimers were characterized by NMR, IR, Gel Permeation Chromatography (GPC), and Matrix Assisted Laser Desorption Ionization-Time of Flight Mass Spectrometry (MALDI-TOF).

The integration of the ^1^H NMR spectra allowed us to determine the actual functionalization and molecular weight of the codendrimers synthesized and the results obtained did not deviate significantly from the expected ones. The values of molecular weight obtained by MALDI-TOF were in reasonably good agreement with those calculated by NMR. GPC and MALDI-TOF afforded useful information about the molecular weight distribution of the polymeric materials under study and, in both cases, a narrow distribution was determined (Table 6).

***Methyl 4-(10-bromodecyloxy)benzoate (1)***

A suspension of potassium carbonate (7.36 g, 53.3 mmol), methyl 4-hydroxybenzoate (3.24 g, 21.3 mmol), 1,10-dibromodecane (6.39 g, 21.3 mmol), and [18]-crown-6 ether (0.75 g, 2.8 mmol) in dry acetone (200 mL) was refluxed for 24 h. The reaction mixture was cooled and filtered off to remove the inorganic salts. The filtrate was washed with dichloromethane and the organic solvents were evaporated. A white solid was obtained, which was purified by column chromatography in silica gel using a mixture of hexanes and ethyl acetate (95/5) as an eluent. Yield: 81%.

**^1^H NMR** (400 MHz, CDCl3) (ppm): 8.01–7.94 (AA’BB’, 2H), 7.93–7.86 (AA’BB’, 2H), 4.00 (t, 2H, J = 6.5 Hz), 3.88 (s, 3H), 3.41 (t, 2H, J = 6.8 Hz), 1.90–1.74 (m, 4H), 1.51–1.28 (m, 12H).

**IR** (KBr), $\tilde{v}$ (cm^−1^): 2921, 2852 (C-H st), 1721 (C=O st), 1607 (C=C st), 1461 (C-H δ), 1278, 1256 (C-O st).

***Methyl 4-(10-azidodecyloxy)benzoate (2)***

First, 0.96 g (14.73 mmol) of sodium azide were slowly added over a stirred solution of compound 1 (0.91 g, 2.46 mmol) and benzyltetraethylammonium chloride (TEBAC) (25 mg, 0.11 mmol) in 25 mL of dry acetone at room temperature. The mixture was refluxed for 24 h, and afterwards, the solvent was removed by rotary evaporation. The crude obtained was dissolved in water and extracted with ethyl ether (2 × 100 mL). The organic phase was dried over anhydrous magnesium sulfate, and the solvent evaporated. A white solid was obtained, which was used without further purification. Yield: 91%.

**^1^H NMR** (400 MHz, CDCl3) (ppm): 8.00–7.94 (AA’BB’, 2H), 7.92–7.86 (AA’BB’, 2H), 4.00 (t, 2H, J = 7.2 Hz), 3.88 (s, 3H), 3.25 (t, 2H, J = 7.2 Hz), 1.84–1.73 (m, 2H), 1.65–1.53 (m, 2H), 1.50–1.28 (m, 12H).

**IR** (KBr), $\tilde{v}$ (cm^−1^): 2921, 2851 (C-H st), 2090 (N3 st), 1716 (C=O st), 1602 (C=C st), 1275, 1251 (C-O st).

***4-(10-azidodecyloxy)benzoic acid (3)***

To a refluxing solution of compound 2 (1.2 g, 3.6 mmol) in methanol (50 mL), an aqueous solution of potassium hydroxide (0.7 g, 12.5 mmol in 10 mL water) was slowly added. The mixture was kept under reflux for 8 h and then cooled down. Hydrochloric acid 2M was added until the solution reached pH 5. Extraction with dichloromethane yielded a white solid, which was used without further purification. Yield: 57%.

**^1^H NMR** (400 MHz, CDCl3) (ppm): 8.09–8.03 (AA’BB’, 2H), 7.96–7.90 (AA’BB’, 2H), 4.02 (t, 2H, J = 6.4 Hz), 3.26 (t, 2H, J = 6.8 Hz), 1.85–1.76 (m, 2H), 1.64–1.55 (m, 2H), 1.51–1.28 (m, 12H).

**IR** (KBr), $\tilde{v}$ cm^−1^): 2922, 2854 (C-H st), 2094 (N3 st), 1677 (C=O st), 1604 (C=C st), 1291, 1260 (C-O st).

***N-succinimidyl 4-(10-azidodecyloxy)benzoate (4)***

To a solution of compound **3** (1.17 g, 3.67 mmol) in dichloromethane (50 mL), 4-dimethylaminopyridine (DMAP) (0.05 g, 0.42 mmol) and N-hydroxysuccinimide (NHS) (0.46 g, 4.04 mmol) were added. The mixture was cooled down to −5 °C and then dicyclohexylcarbodiimide (DCC) (0.83 g, 4.04 mmol) was slowly added. After 24 h stirring at room temperature, the reaction mixture was filtered off and the orange solid obtained was purified by column chromatography in silica gel using a mixture of hexanes and ethyl acetate (95/5) as an eluent. Yield: 90%.

**^1^H NMR** (400 MHz, CDCl_3_) δ (ppm): 8.09–8.04 (AA’BB’, 2H), 7.97–7.92 (AA’BB’, 2H), 4.03 (t, 2H, *J* = 6.8 Hz), 3.25 (t, 2H, *J* = 6.8 Hz), 2.88 (bs, 4H), 1.84–1.76 (m, 2H), 1.63–1.55 (m, 2H), 1.50–1.28 (m, 12H).

**^13^C NMR** (100 MHz, CDCl_3_) δ (ppm): 169.45, 164.47, 161.44, 132.80, 116.72, 114.58, 68.39, 51.41, 29.34, 29.32, 29.22, 29.06, 28.93, 28.77, 26.64, 25.85, 25.63.

**IR** (KBr), $\tilde{v}$ (cm^−1^): 2922, 2854 (C-H st), 2094 (N_3_ st), 1677 (C=O st), 1604 (C=C st), 1291, 1260 (C-O st).

**MS** (HR-ESI^+^) *m*/*z*: Calculated for C_21_H_28_N_4_O_5_: 416.2 [M]^+^. Found: 439.2 [M + Na]^+^.

**Elemental analysis:** Calculated for C_21_H_28_N_4_O_5_: C, 60.56%; H, 6.78%; N, 13.45%. Found: C, 60.59%; H, 6.99%; N, 13.47%.

***Methyl 4-(11-hydroxyundecyloxy)benzoote (5)***

In a round-bottom flask, methyl 4-hydroxybenzoate (3.89 g, 25.5 mmol), potassium carbonate (8.80 g, 63.7 mmol), 18-crown-6 ether (0.80 g, 3.03 mmol), and 11-bromoundecanol (6.54 g, 25.5 mmol) were dissolved in dry acetone (200 mL). The mixture was refluxed for 24 h, cooled down to room temperature, and filtered off through paper. The organic solvent was removed by rotary evaporation and the white solid obtained was recrystallized from methanol. Yield: 85%.

**^1^H NMR** (400 MHz, CDCl_3_) δ (ppm): 7.99–7.94 (AA’BB’, 2H), 6.91–6.84 (AA’BB’, 2H), 3.97 (t, 2H, *J* = 6.6 Hz), 3.85 (s, 3H), 3.60 (t, 2H, *J* = 6.6 Hz), 1.82–1.71 (m, 2H), 1.59–1.48 (m, 2H), 1.48–1.24 (m, 14H).

**IR** (KBr), $\tilde{v}$ (cm^−1^): 3311 (O-H st), 2921, 2851 (C-H st), 1722 (C=O st), 1607 (C=C st), 1280, 1257 (C-O st).

***Methyl 4-[11-(prop-2-ynyloxy)undecyloxy]benzoate (6)***

Over 3 g (9.3 mmol) of compound **5**, an 80%-wt. solution of propargyl bromide in toluene (20.8 g, 139.6 mmol) and tetrabutylammonium iodide (TBAI) (1.63 g, 4.4 mmol) were added. Then, a 60%-wt. aqueous solution of sodium hydroxide (10.2 mL) was added dropwise. The reaction was maintained under argon, at room temperature and during 5 h. The mixture was carefully poured onto 50 mL of hydrochloric acid 2M, and then extracted three times with methylene chloride. The combined organic layers were washed with water and brine. Concentration by rotary evaporation yielded a white solid, which was further purified by column chromatography in silica gel using a mixture of hexanes and ethyl acetate (95/5) as an eluent. Yield: 41%.

**^1^H NMR** (400 MHz, CDCl_3_) δ (ppm): 7.99–7.95 (AA’BB’, 2H), 6.92–6.88 (AA’BB’, 2H), 4.13 (d, 2H, *J* = 2.2 Hz), 4.00 (t, 2H, *J* = 6.6 Hz), 3.88 (s, 3H), 3.50 (t, 2H, *J* = 6.6 Hz), 2.41 (t, 1H, *J* = 2.2 Hz), 1.83–1.74 (m, 2H), 1.63–1.55 (m, 2H), 1.49–1.40 (m, 2H), 1.39–1.26 (m, 12H).

**IR** (KBr), $\tilde{v}$ (cm^−1^): 3275 (≡C-H st), 2921, 2851 (C-H st), 1721 (C=O st), 1605 (C=C st), 1462 (C-H δ), 1279, 1252 (C-O st).

***4-[11-(prop-2-ynyloxy)undecyloxy]benzoic acid (7)***

First, 0.81 g (2.25 mmol) of compound **6** were dissolved in methanol (50 mL). The mixture was refluxed and then an aqueous solution of potassium hydroxide (0.6 g, 10 mL water) was added dropwise. The reaction mixture was refluxed for 7 h, and then cooled down. Hydrochloric acid 2M was added to acidify the mixture (pH 1). The product was extracted with dichloromethane (3 × 25 mL). The combined organic layers were washed with brine. After removal of the solvent by rotary evaporation, a white solid was obtained, which was used without further purification. Yield: 98%.

**^1^H NMR** (400 MHz, DMSO-d_6_) δ (ppm): 12.60 (s, 1H), 7.89–7.84 (AA’BB’, 2H), 7.01–6.97 (AA’BB’, 2H), 4.08 (d, 2H, *J* = 2.4 Hz), 4.02 (t, 2H, *J* = 6.4 Hz ), 3.40 (t, 2H, *J* = 2.4), 3.39 (t, 1H, *J* = 6.4 Hz) 1.75–1.67 (m, 2H), 1.53–1.48 (m, 2H), 1.43–1.36 (m, 2H), 1.35–1.26 (m, 12H).

**IR** (KBr), $\tilde{v}$ (cm^−1^): 3269 (O-H st), 2920, 2851 (C-H st), 1669 (C=O st), 1607 (C=C st), 1461 (C-H δ), 1253 (C-O st).

***N-succinimidyl 4-[11-(prop-2-ynyloxy)undecyloxy]benzoate (8)***

To a solution of compound **7** (0.72 g, 2.2 mmol) in dichloromethane (50 mL), DMAP (0.03 g, 0.25 mmol) and NHS (0.46 g, 2.42 mmol) were added. The mixture was cooled to −5 °C and DCC (0.50 g, 2.42 mmol) was slowly added. After 24 h stirring at room temperature, the reaction mixture was filtered off and the white solid obtained was purified by column chromatography in silica gel using a mixture of hexanes and ethyl acetate (9/1) as an eluent. Yield: 95%.

**^1^H NMR** (400 MHz, CDCl_3_) δ (ppm): 8.09–8.04 (AA’BB’, 2H), 6.97–6.92 (AA’BB’, 2H), 4.13 (d, 2H, *J* = 2.4 Hz), 4.03 (t, 2H, *J* = 6.8 Hz), 3.50 (t, 2H, *J* = 6.4 Hz), 2.89 (bs, 4H), 2.41 (t, 1H, *J* = 2.4 Hz), 1.84–1.76 (m, 2H), 1.63–1.55 (m, 2H), 1.49–1.41 (m, 2H), 1.39–1.26 (m, 12H).

**^13^C NMR** (100 MHz, CDCl_3_) δ (ppm): 169.49, 164.57, 161.53, 132.89, 116.78, 114.66, 80.09, 74.06, 70.33, 68.49, 58.03, 29.54, 29.52, 29.50, 29.44, 29.36, 29.02, 26.11, 25.95, 25.71.

**IR** (KBr), $\tilde{v}$ (cm^−1^): 3261 (O-H st), 2917, 2850 (C-H st), 1765, 1746 (C=O st), 1577 (C=C st), 1462 (C-H δ), 1259, 1243, 1211, 1171 (C-O st).

**MS** (MALDI^+^, dithranol) *m*/*z*: Calculated for C_25_H_33_NO_6_: 443.2 [M]^+^. Found: 466.2 [M + Na]^+^.

**Elemental analysis:** Calculated for C_25_H_33_NO_6_: C, 67.70%; H, 7.50%; N, 3.16%. Found: C, 67.74%; H, 7.87%; N, 3.54%.

***N-succinimidyl 4-(butoxy)benzoate (9)***

To a solution of 4-butoxybenzoic acid (5.0 g, 26 mmol) in dry dichoromethane (25 mL), DMAP (0.39 g, 3.2 mmol) and NHS (3.22 g, 28 mmol) were added. The mixture was cooled to −5 °C and then DCC (6.37 g, 31 mmol) was slowly added. After 48 h stirring at room temperature, the reaction mixture was filtered off and the white solid obtained was purified by column chromatography in silica gel using a mixture of hexanes and ethyl acetate (1/1) as an eluent. Yield: 83%.

**^1^H NMR** (400 MHz, CDCl_3_) δ (ppm): 8.10–8.05 (AA’BB’, 2H), 6.98–6.93 (AA’BB’, 2H), 4.04 (t, 2H, *J* = 6.6 Hz), 2.90 (bs, 4H), 1.84–1.75 (m, 2H), 1.55–1.46 (m, 2H), 0.99 (t, 2H, *J* = 7.4 Hz).

**^13^C NMR** (100 MHz, CDCl_3_) δ (ppm): 169.49, 164.45, 161.43, 132.74, 116.63, 114.55, 68.03, 30.91, 25.58, 19.04, 13.70.

**IR** (KBr), $\tilde{v}$ (cm^−1^): 2958, 2850 (C-H st), 1764, 1740 (C=O st), 1604, 1511 (C=C st), 1426, 1367 (C-H δ), 1257, 1243, 1204, 1171 (C-O st).

**MS** (MALDI^+^, dithranol) *m*/*z*: Calculated for C_21_H_29_NO_5_: 291.1 [M]^+^. Found: 314.1 [M + Na]^+^.

**Elemental analysis:** Calculated for C_21_H_29_NO_5_: C, 61.85%; H, 5.88%; N, 4.81%. Found: C, 62.11%; H, 6.01%; N, 4.93%.

***N-succinimidyl 4-(decyloxy)benzoate (10)***

To a solution of 4-decyloxybenzoic acid (6.0 g, 21 mmol) in dry dichloromethane (25 mL), DMAP (0.33 g, 2.7 mmol) and NHS (2.76 g, 24 mmol) were added. The mixture was cooled to −5 °C and DCC (5.33 g, 26 mmol) was slowly added. After 48 h stirring at room temperature, the reaction mixture was filtered off and the white solid obtained was purified by column chromatography in silica gel using a mixture of hexanes and ethyl acetate (6/4) as an eluent. Yield: 79%.

**^1^H NMR** (400 MHz, CDCl_3_) δ (ppm): 8.09–8.05 (AA’BB’, 2H), 6.97–6.93 (AA’BB’, 2H), 4.03 (t, 2H, *J* = 6.6 Hz), 2.89 (bs, 4H), 1.84–1.76 (m, 2H), 1.50–1.42 (m, 2H), 1.38–1.24 (m, 12H), 0.88 (t, 3H, *J* = 6.6 Hz).

**^13^C NMR** (100 MHz, CDCl_3_) δ (ppm): 169.53, 164.58, 161.54, 132.88, 116.77, 114.67, 68.49, 31.91, 29.56, 29.36, 29.33, 29.02, 25.95, 25.71, 22.70, 14.14.

**IR** (KBr), $\tilde{v}$ (cm^−1^): 3261 (O-H st), 2917, 2850 (C-H st), 1765, 1746 (C=O st), 1577 (C=C st), 1462 (C-H δ), 1259, 1243, 1211, 1171 (C-O st).

**MS** (MALDI^+^, dithranol) *m*/*z*: Calculated for C_21_H_29_NO_5_: 375.2 [M]^+^. Found: 398.2 [M + Na]^+^.

**Elemental analysis:** Calculated for C_21_H_29_NO_5_: C, 67.18%; H, 7.79%; N, 3.73%. Found: C, 67.27%; H, 7.52%; N, 3.84%.

General Procedure for the Synthesis of the PPI-Derived Codendrimers I and II

First, 0.2 g (56.5 μmol) of G4-PPI dissolved in dry dichloromethane (10 mL) and freshly distilled triethylamine (100 μL) were stirred in a round-bottom flask. Then, a mixture of **9** or **10** (1.65 mmol) and **4** or **8** (171 μmol) was added. Reaction was maintained in the dark, at room temperature, and under argon for 48 h. The solvent was removed by rotary evaporation and the crude obtained was purified by column chromatography in silica gel using dichloromethane as an eluent. A pale yellow solid was obtained in 72–85% yield.

***Codendrimer I***

**^1^H NMR** (400 MHz, CDCl_3_) δ (ppm): 8.20–7.92 (H_k_, H_v_, (N*H*-CO), 32H), 7.92–7.75 (H_a_,H_a’_,H_c_,H_c’_, 64H), 6.82–6.57 (H_b_,H_b’_,H_d_,H_d’_, 64H), 4.12 (H_m_, d, 6H, *J* = 2.4 Hz), 3.86 (t, H_e_,H_f_ (C*H*_2_-O), 64H, *J* = 6.4 Hz), 3.53–3.36 (H_i_ + C*H*_2_-NH-CO (PPI), 70H), 2.65–2.25 (C*H*_2_N_amino_ (PPI) + H_o_, 183H), 1.80–1.18 (H_g_, H_h_ + NCH_2_C*H*_2_CH_2_N (PPI), 642H), 0.87 (H_l_, t, 87H, *J* = 7.4 Hz).

**^13^C NMR** (100 MHz, CDCl_3_) δ (ppm): 167.37 (C_r_, C_s_), 161.50 (C_u_, C_p_), 129.06 (C_a_, C_c_), 126.55 (C_t_, C_q_), 113.92 (C_b_, C_d_,), 74.04 (C_n_), 70.55 (C_i_), 70.27 (C_e_), 68.07 (C_f_), 57.98 (C_m_), 52.15, 51.68 (*C*H_2_-N_amino_ (PPI)), 38.53 (*C*H_2_-NHCO), 31.17 (*C*H_2_CH_3_), 29.69, 29.60, 29.47, 29.33, 29.22, 26.04 (C_h_, C_g_), 27.10 (NCH_2_*C*H_2_CH_2_N (PPI)), 22.67 (*C*H_2_CH_2_CH_3_), 14.12 (C_l_).

**IR** (KBr) $\tilde{v}$ (cm^−1^): 3500–3200 (N-H st), 3314 (≡C-H st), 2924, 2854 (C-H st), 1635 (C=O st), 1608, 1574, 1541, 1506 (arC=C), 1468 (C-H δ), 1300, 1252, 1180 (C-O st).

**MS** (MALDI^+^, dithranol) *m*/*z*: Calculated for C_740_H_1212_N_62_O_67_: 12,049.4 [M]^+^ (100%). Found: 11,984.1 (100%), 11,915.5 (88.2%), 12,051.6 (88.6).

***Codendrimer II***

**^1^H NMR** (400 MHz, CDCl_3_) δ (ppm): 8.10–7.92 (H_k_, H_v_, (N*H*-CO), 32H), 7.90–7.75 (H_a_,H_a’_,H_c_,H_c’_, 64H), 6.83–6.70 (H_b_,H_b’_,H_d_,H_d’_, 64H), 3.88 (t, H_e_,H_f_ (C*H*_2_-O), 64H, *J* = 6.4 Hz), 3.52–3.38 (C*H*_2_-NH-CO (PPI), 64H), 3.24 (H_i_, t, 6H, *J* = 6.8 Hz), 2.50–2.26 (C*H*_2_N_amino_ (PPI), 180H), 1.78–1.62 (NCH_2_C*H*_2_CH_2_N (PPI), 124H), 1.62–1.24 (H_g_, H_h_, H_j_, 164H), 0.95 (H_l_, t, 87H, *J* = 7.4 Hz).

**^13^C NMR** (100 MHz, CDCl_3_) δ (ppm): 167.37 (C_r_, C_s_), 161.48 (C_p_, C_u_), 129.01 (C_a_, C_c_), 126.59 (C_t_, C_q_), 113.92 (C_b_, C_d_), 67.71 (C_f_), 52.29, 52.12, 51.71 (*C*H_2_-N_amino_ (PPI)), 51.42 (C_i_), 38.62 (*C*H_2_-NHCO), 31.17 (C_j_), 29.42, 29.40, 29.37, 29.17, 29.11, 28.80, 26.67, 25.98 (C_g_), 27.03 (NCH_2_*C*H_2_CH_2_N (PPI)), 19.17 (C_h_), 13.83 (C_l_).

**IR** (KBr) $\tilde{v}$ (cm^−1^): 3323 (N-H st), 2934, 2871 (C-H st), 2095 (N_3_ st), 1635 (C=O st), 1607, 1541, 1506 (arC=C), 1471 (C-H δ), 1305, 1252, 1180 (C-O st).

**MS** (MALDI^+^, dithranol) *m*/*z*: Calculated for C_554_H_849_N_71_O_64_: 9527.6 [M]^+^ (100%). Found: 9300.4 [M + Na]^+^ (100%).

The average relative composition of both peripheral units (4-alkyloxyphenyl, and azide- or alkyne-terminated ones) in the final codendrimer as determined by NMR was quite similar to the expected one. The values of Mn and Mw obtained by MALDI-TOF were in reasonably good agreement with the calculated values by NMR. Then, as expected, the GPC measurements underestimated the Mn and Mw values as a consequence of the calibration of the apparatus with standards of polystyrene [38]. However, this technique affords a useful information about the molecular weight distribution of the polymeric material under study and, in both cases, a narrow distribution has been determined.

### 3.2. Preparation of the Dendrimeric Network

In a 26 mm-diameter glass vial, 151 mg (12.5 μmol) of codendrimer I and 119 mg (12.5 μmol) of codendrimer II were dissolved in 1 mL of dry THF using ultrasounds. In another vial, 1.9 mg (7.5 μmol) of copper(II) sulfate pentahydrate and 3.0 mg (15 μmol) of sodium ascorbate were dissolved in 250 μL of water. This solution was poured with gentle stirring onto the codendrimers solution. The vial was closed and mechanically stirred at 160 rpm for 24 h at room temperature. The reagents mixture affords a consistent macromolecular gel. After the reaction time established, 3 mL of water were added and decanted. The network formed was obtained as a pellet which was carefully removed from the vial bottom. Unreacted codendrimers and other low molecular weight by-products were removed by washing the network with THF (3 times). Finally, the network was dried at room temperature.

### 3.3. General Method for the Encapsulation/Release of Dyes

A sample of the dry network was weighed and immersed in a solution of the dye in the appropriate solvent of a known concentration. After 24 h, the solution was removed and the swollen network was weighed, dried, and weighed again. Once the network containing the dye was dried, it was immersed in the corresponding solvent, so that the release of the dye occurs. The amount of dye released from the network was determined by UV-vis spectroscopy, by interpolation in the corresponding calibration curves. In the case of Rhodamine B, the calibration curve was obtained using four solutions of Rh-B in ethanol, the pH of which was adjusted to approx. 7.5 with triethylamine. For Disperse red 1 and β-Carotene, four solutions of the dye in tetrahydrofuran were used. The concentration of the dye solution in the network was easily calculated knowing the amount of dye released and the weight (volume) of the solvent adsorbed by the network.

### 3.4. Techniques

NMR experiments (^1^H, ^13^C) were recorded using standard pulse sequences on AVANCE 400 spectrometer operating at 400 MHz for ^1^H and 100 MHz for ^13^C.

Elemental analysis (EA) was performed using a Perkin-Elmer 240C microanalyzer.

Gel Permeation Chromatography (GPC) was performed in an equipment constituted by a pump (Waters 600), a controller and a separation module (Waters e2695). The stationary phase was Ultrastyragel and the columns were of 19 × 300 mm^2^ and 10^4^ Å and 500 Å, respectively. Double detection of light scattering and UV-vis was used. Calibration was performed using polystyrene (PS) standards. Samples of 8 μL were injected and the solvent was tetrahydrofuran (THF) with 1% of triethylamine.

IR spectra were obtained with a Nicolet Avatar 360 FTIR spectrophotometer in the 400–4000 cm^−1^ spectroscopic range.

The optical textures of the mesophases were studied with a Nikon polarized-light optical microscope equipped with a Linkam THMS 600 hot-stage and a Linkam TMS 91 central processor and with an Olympus polarized-light optical microscope BX51 equipped with a Linkam hot-stage and Linkam TMS 91 central processor.

The transition temperatures and enthalpies were measured by Differential Scanning Calorimetry (DSC) with TA Q20 and Q2000 Instruments operated at a scanning rate of 10 °C min^−1^ on both heating and cooling. The apparatus were calibrated with indium.

Thermogravimetric analysis was carried out in a TGA-Q5000 apparatus at a heating rate of 10°/min under nitrogen up to 600 °C and under air from 600 to 750 °C.

The XRD patterns were obtained with a pinhole camera (Anton-Paar) operating with a point-focused Ni-filtered Cu-Kα beam. The sample was held in Lindemann glass capillaries (1 mm diameter) and heated, when necessary, with a variable-temperature oven. The capillary axis is perpendicular to the X-ray beam and the pattern is collected on a flat photographic film perpendicular to the X-ray beam. Distances were obtained via Bragg’s law.

SEM experiments were carried out in a JEOL JSM6400 apparatus. Samples were placed on quartz plates and covered with gold.

The uniaxial tensile assays (stress-strain curves) were performed in an INSTRON 5548 and a 5848 MicroTester apparatus, using a 50 N charge cell with a video extensometer INSTRON 2663–281 at room temperature.

UV-vis spectrometer ATI-Unicam UV4-200.

Fluorimeter PERKIN ELMER LS50B, with 10 mm quartz cuvettes for liquid samples and quartz plates for solid samples.

## 4. Conclusions

A liquid crystal polymer network has been prepared by the crosslinking of two reactive codendrimers via a dipolar 1,3-cycloaddition of azides and alkynes. This is the first system of these characteristics described up to date, and shows a variety of properties, including liquid crystallinity, viscoelasticity, porosity, and the ability to selectively swell with organic solvents and encapsulate small molecules, like organic dyes. The careful selection of the structural features of the dendrimeric systems involved in the polymeric network (including diverse functional units, size, chemical nature, mesophase type, number of reactive crosslinking units, etc.) provides a powerful tool for the preparation of materials with highly tunable mechanical and optical properties, and therefore, it opens a path full of possibilities in the design and achievement of multifunctional soft materials with potential applications in fields like sensors, mechanical actuators, lasers, catalysis, drug delivery, etc.

## Figures and Tables

**Table 1:** Thermal properties and XRD data of the codendrimers I and II and the dendrimeric network.

Compound	Phase Transitions ^a,b,c^	hkl ^e^	d_exp_^f^ (Å)	d_calc_^f^ (Å)	σ ^g^ (Å)
Codendrimer I	g 24 SmA 60 (0.9) I	001	45.3	44.9	44.9
		002	22.2	22.5	
Codendrimer II	g 25 I				
Network	g 20 SmA 57 ^d^ I	001	42.2	42.4	42.4
		002	21.3	21.2	

[^a^] g = glass, SmA = smectic A mesophase, I = isotropic liquid. [^b^] DSC temperatures in °C. [^c^] In brackets, transition enthalpy in J/g. [^d^] Temperature determined by polarized optical microscopy (POM). [^e^] hkl: Miller indexes. [^f^] d_exp_: Bragg reflections distance measured experimentally; d_calc_: Bragg reflections distance calculated from σ parameter. [^g^] σ parameter: smectic layer thickness, calculated from the experimental Bragg reflections distances.

**Table 2:** Results for the uniaxial tensile assays.

	Young Module (MPa)	Elastic Deform. (%)	Max. Charge (MPa)	Max. Deform. (%)
**Network**	17.4	5	12.7	159.8

**Table 3:** Swelling ratio of the dendrimeric network for several solvents.

Solvent	Network wt (mg) [Wd]	Swollen Network wt (mg) [Ws + Wd]	Swelling Ratio [SR = Ws/Wd]
Dichloromethane	8.2	88.5	9.8
Tetrahydrofuran	3.8	36.5	8.6
Ethanol	8.1	24.4	2.0
Water	11.1	21.4	0.9
n-Hexane	13.5	20.8	0.5

**Table 4:** Encapsulation/release experiments of Rhodamine B in the network.

Network Wt. (mg)	Rh-B/EtOH Concentration	Swollen Network (mg)	Rh-B Desorbed ^a^ (μg)	Rh-B/EtOH (in the Network) Concentration
31.6	1.25 × 10^−4^ M	75.7	8.3	3.1 × 10^−4^ M
29.9	1.0 × 10^−3^ M	72.2	68.5	2.6 × 10^−3^ M
29.5	1.0 × 10^−2^ M	68.1	584	2.5 × 10^−2^ M

^a^ Calibration curve constructed from UV-vis measurements of solutions of Rhodamine B in ethanol (pH 7.3, λ_a_ = 543 nm).

**Table 5:** Results of the experiments of encapsulation/release of Rh-B, DR1 and βC in the dendrimeric network.

	Initial Dye/Solvent Concentration	Dye Desorbed ^a^ (μg)	Dye/Solvent (in the Network) Concentration
Rh-B/EtOH	1.25 × 10^−4^ M	8.3	3.1 × 10^−4^ M
DR1/THF	9.32 × 10^−5^ M	5.9	10.5 × 10^−5^ M
bC/THF	2.68 × 10^−4^ M	8.0	2.21 × 10^−4^ M

^a^ Calibration curves constructed from UV-vis measurements of solutions of Rhodamine B in ethanol (pH 7.3, λ_a_ = 543 nm), Disperse Red 1 in THF (λ_a_ = 486 nm), and β-Carotene in THF ((λ_a_ = 460 nm).

**Table 6:** Molecular weights and polydispersity index of codendrimers I and II as determined by NMR, MALDI, and GPC.

Compound	Theoretical ^a^	NMR ^b^		MALDI			GPC ^c^	
	Mn (Ratio)	Mn (Ratio)	Mn	Mw	PDI ^d^	Mn	Mw	PDI ^d^
Codendrimer I	12,050 (29/3)	11,790 (28.5/2.6)	11,737	11,744	1.001	5728	6258	1.09
Codendrimer II	9528 (29/3)	9555 (27.6/3.9)	8949	8922	1.003	4109	4743	1.15

^a^ Considering the final composition of peripheral units as charged in the synthetic procedure. ^b^ Considering the experimental composition of peripheral units on average as determined by integration of the NMR signals. The integration of the signal corresponding to C*H*_2_-N_3_ is overestimated since it is close to the signal corresponding to C*H*_2_NHCO-. ^c^ Calibrated with Polystyrene standards. ^d^ *Stricto sensu* this value indicates the molecular weight distribution of the materials since they are not formed by the polymerization of repeating units but by the functionalization of a dendrimer.
